# Correction: *TP53* exon-6 truncating mutations produce separation of function isoforms with pro-tumorigenic functions

**DOI:** 10.7554/eLife.25532

**Published:** 2017-02-01

**Authors:** Nitin H Shirole, Debjani Pal, Edward R Kastenhuber, Serif Senturk, Joseph Boroda, Paola Pisterzi, Madison Miller, Gustavo Munoz, Marko Anderluh, Marc Ladanyi, Scott W Lowe, Raffaella Sordella

Shirole NH, Pal D, Kastenhuber ER, Senturk S, Boroda J, Pisterzi P, Miller M, Munoz G, Anderluh M, Ladanyi M, Lowe SW, Sordella R. 2016. *TP53* exon-6 truncating mutations produce separation of function isoforms with pro-tumorigenic functions. *eLife* **5**:e17929. doi: 10.7554/eLife.17929.

Published 19, October 2016

We identified an error in figure 3F with the labelling of cell lines. The error might have appeared from figure 5C which has a very similar outline for labelling the cell lines. We have provided the corrected figure 3 below.

The corrected figure 3 is shown here:

**Figure:**
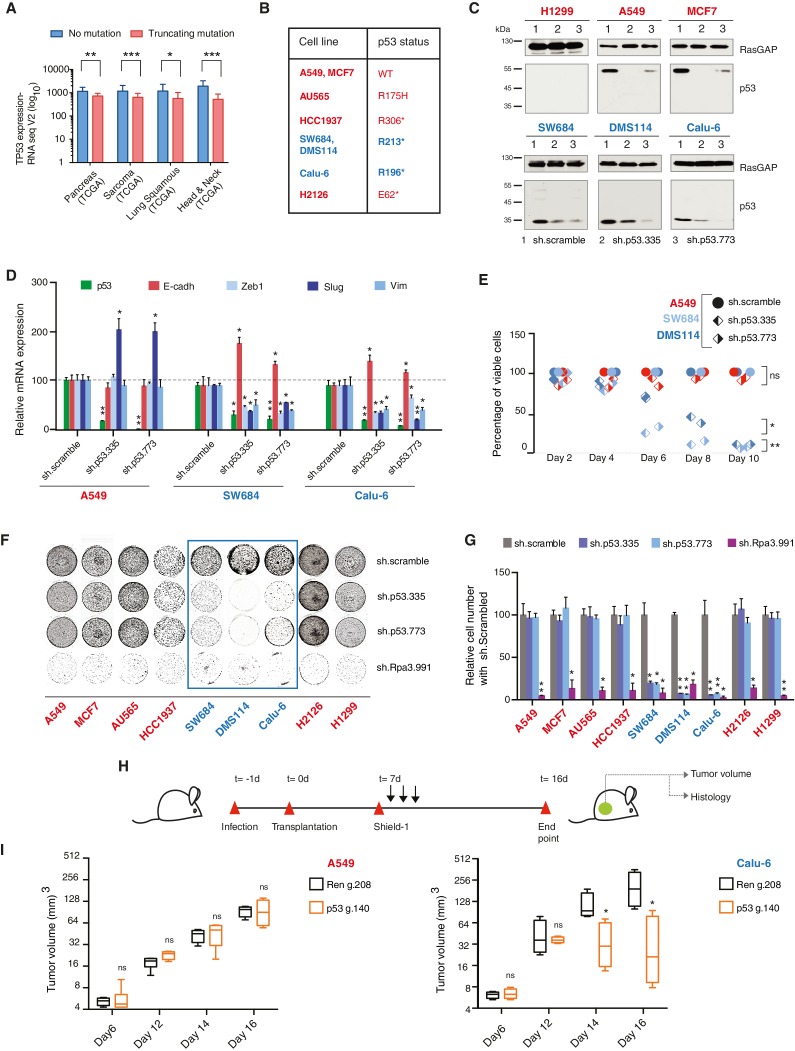

The originally published figure 3 is also shown for reference:

**Figure:**
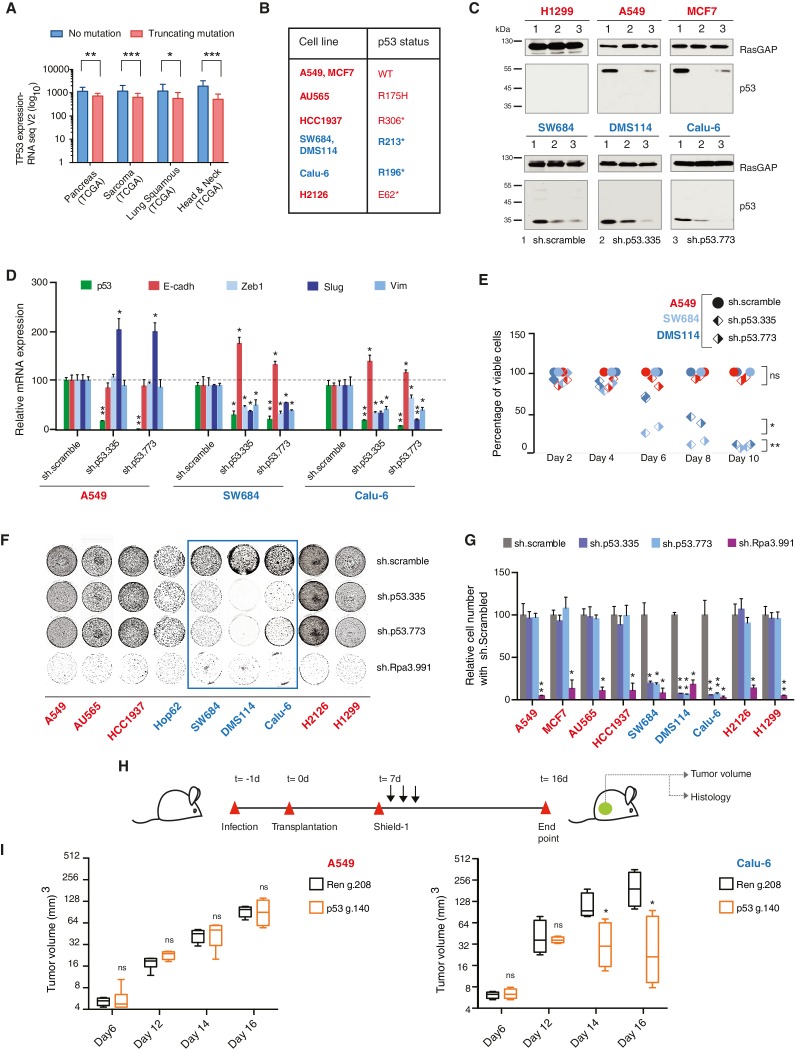

The article has been corrected accordingly.

