# Supplementary material for: TP53 exon-6 truncating mutations produce separation of function isoforms with pro-tumorigenic functions
Source: eLife. 2016 Oct 19;5:e17929. doi: 10.7554/eLife.17929 (PMC5092050; doi:10.7554/eLife.17929)
Supplement: Supplementary file 3. — DOI: http://dx.doi.org/10.7554/eLife.17929.032 [file elife-17929-supp3.docx]

**Supplementary File 3:** Number of tumor samples with indicated mutation types in primary and metastatic colorectal carcinoma.

| Mutation Type | Primary CRC | Metastatic CRC | Ratio |
| --- | --- | --- | --- |
| Splice | 11 | 23 | 2.090909091 |
| Ex6 NS | 12 | 24 | 2 |
| Other NS | 22 | 22 | 1 |
| IF indel | 3 | 10 | 3.333333333 |
| FS indel | 32 | 29 | 0.90625 |
| MS | 170 | 175 | 1.029411765 |
| Multiple | 21 | 19 | 0.904761905 |
| None | 130 | 92 | 0.707692308 |
