# Supplementary material for: TP53 exon-6 truncating mutations produce separation of function isoforms with pro-tumorigenic functions
Source: eLife. 2016 Oct 19;5:e17929. doi: 10.7554/eLife.17929 (PMC5092050; doi:10.7554/eLife.17929)
Supplement: Supplementary file 4. — DOI: http://dx.doi.org/10.7554/eLife.17929.033 [file elife-17929-supp4.docx]

**Supplementary File 4:** Sequence of sense strand of shRNAs used in this study.

| shRNA | Sequence |
| --- | --- |
| Sh.p53-335 | 5'- CGGCGCACAGAGGAAGAGAAT -3' |
| Sh.p53-773 | 5'- GAAGACTCCAGTGGTAATCTA -3' |
| Sh.CypD-2682 | 5'- GTTCTTCATCTGCACCATAAA -3' |
| Sh.CypD-2683 | 5'- ATAGAATCTTTCGGCTCTAAG -3' |
| Sh.Rpa3-991 | 5'- GATCTTGGACTTTACAATGAA -3' |
