# Supplementary material for: TP53 exon-6 truncating mutations produce separation of function isoforms with pro-tumorigenic functions
Source: eLife. 2016 Oct 19;5:e17929. doi: 10.7554/eLife.17929 (PMC5092050; doi:10.7554/eLife.17929)
Supplement: Supplementary file 5. — DOI: http://dx.doi.org/10.7554/eLife.17929.034 [file elife-17929-supp5.docx]

Supplementary File 5: Complimentary oligonucleotides used for cloning the indicated sgRNAs.

| sgRNA | Sequence |
| --- | --- |
| Ren g.208-F | 5'- CACCgGGTATAATACACCGCGCTAC -3' |
| Ren g.208-R | 5'- AAACGTAGCGCGGTGTATTATACCc -3' |
| p53 g.13-F | 5'- CACCgTCGACGCTAGGATCTGACTG -3' |
| p53 g.13-R | 5'- AAACCAGTCAGATCCTAGCGTCGAc -3' |
| p53 g.140-F | 5'- CACCgCCATTGTTCAATATCGTCCG -3' |
| p53 g.140-R | 5'- AAACCGGACGATATTGAACAATGGc -3' |
| CypD g.131-F | 5'- CACCgCAGGTACACGAGCGGGTTCC -3' |
| CypD g.131-R | 5'- AAACGGAACCCGCTCGTGTACCTGc -3' |
| Rpa3 g.44-F | 5'- CACCgGATGAATTGAGCTAGCATGC -3' |
| Rpa3 g.44-R | 5'- AAACGCATGCTAGCTCAATTCATCc -3' |
